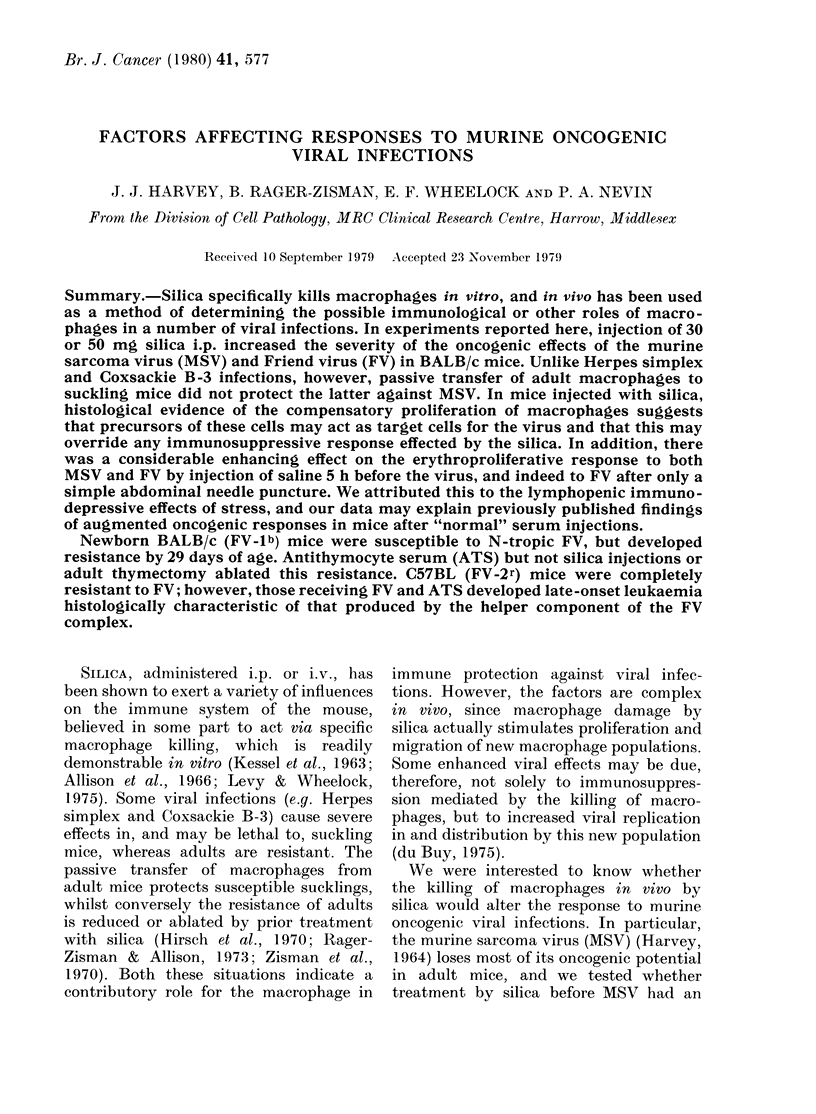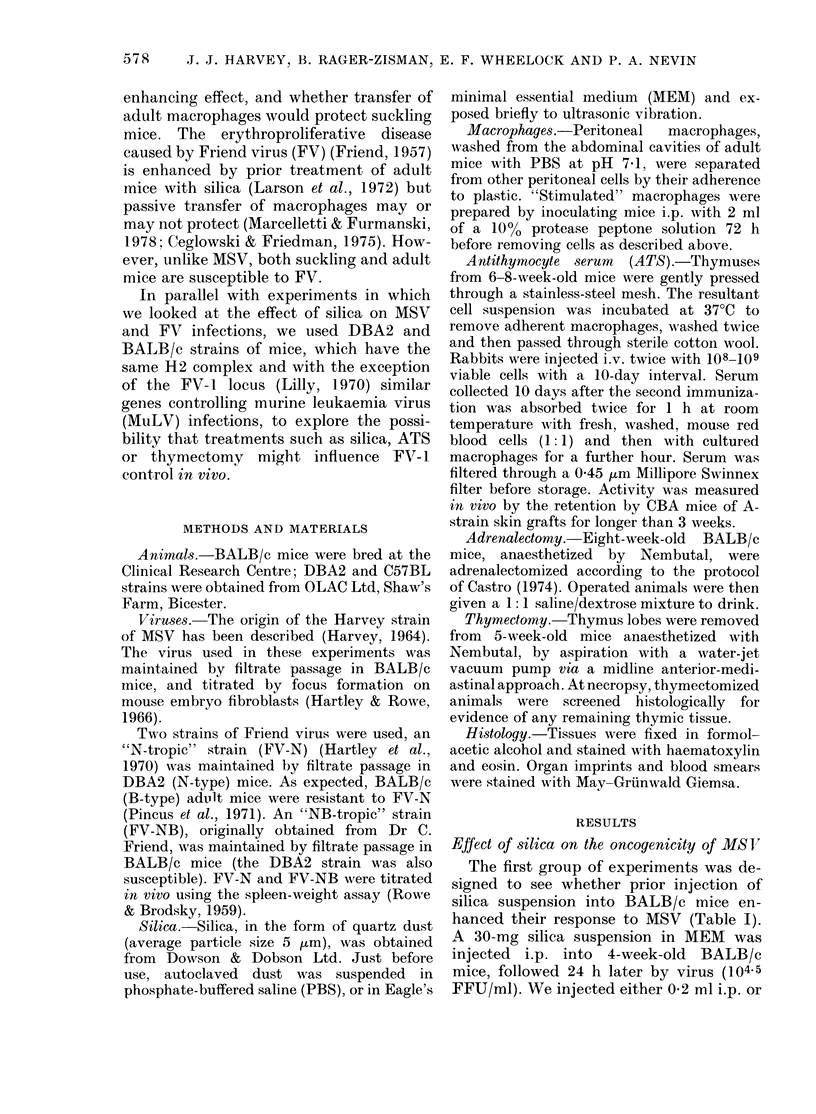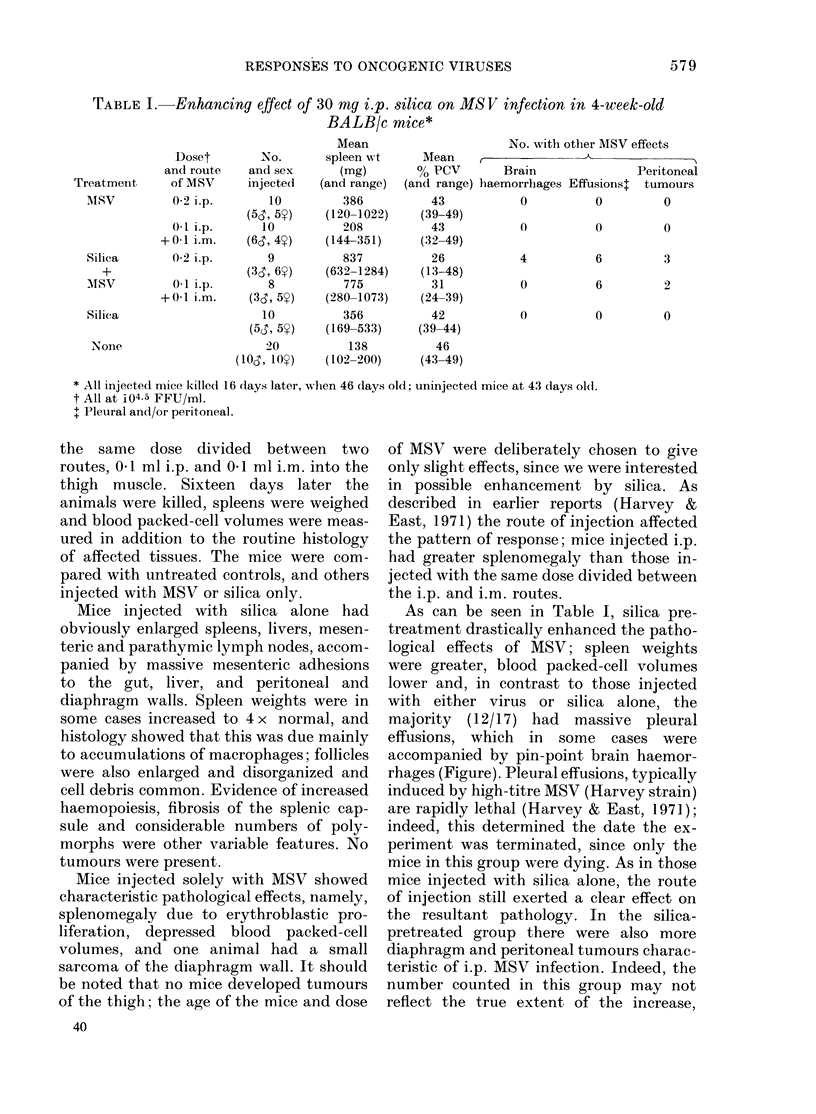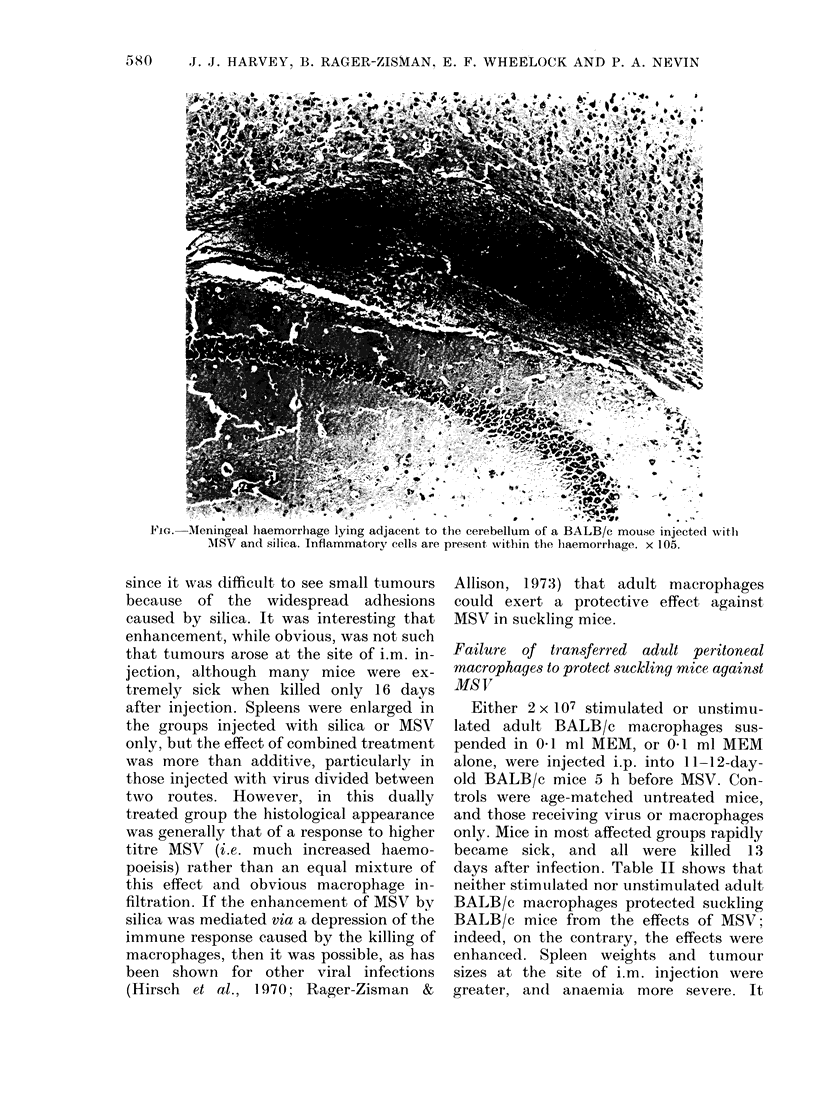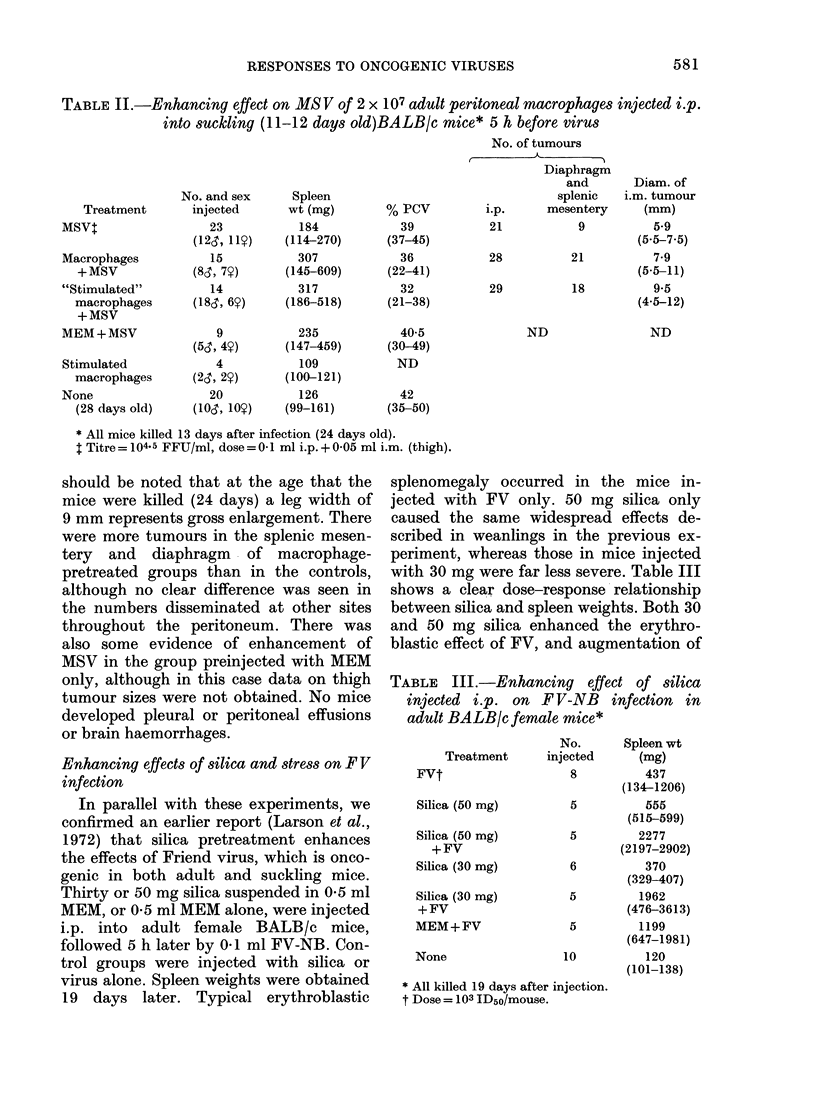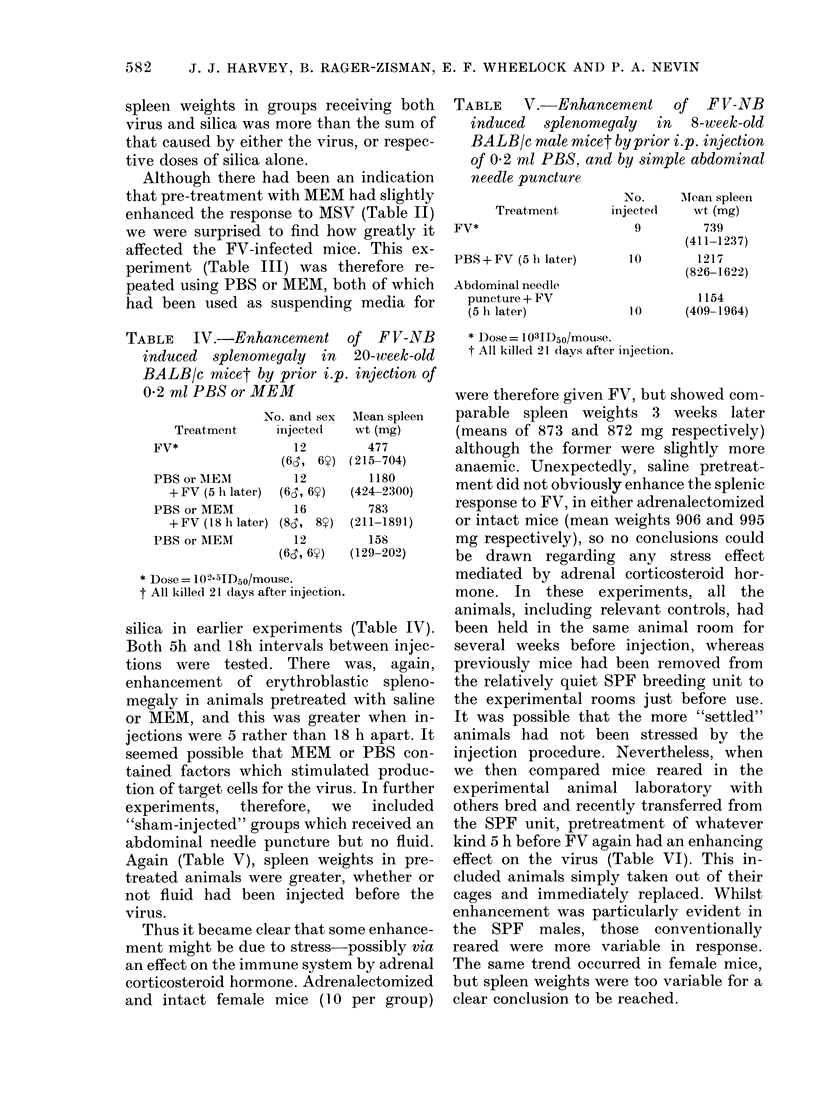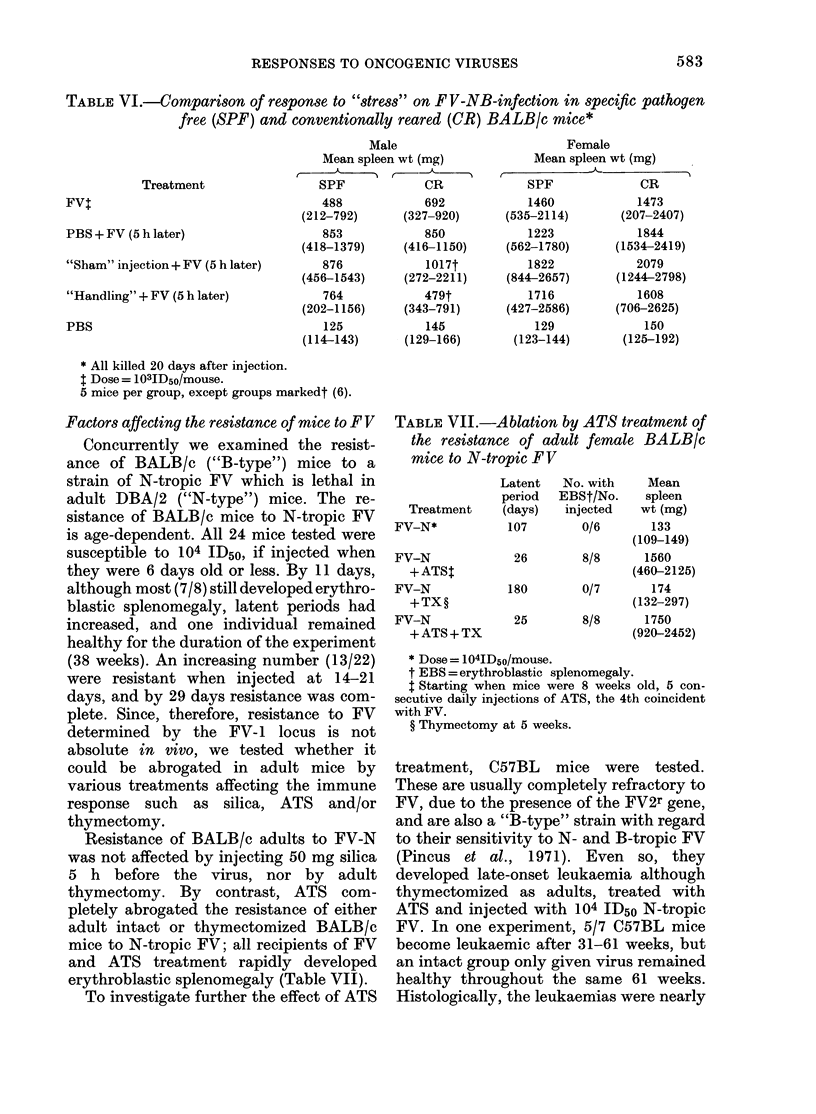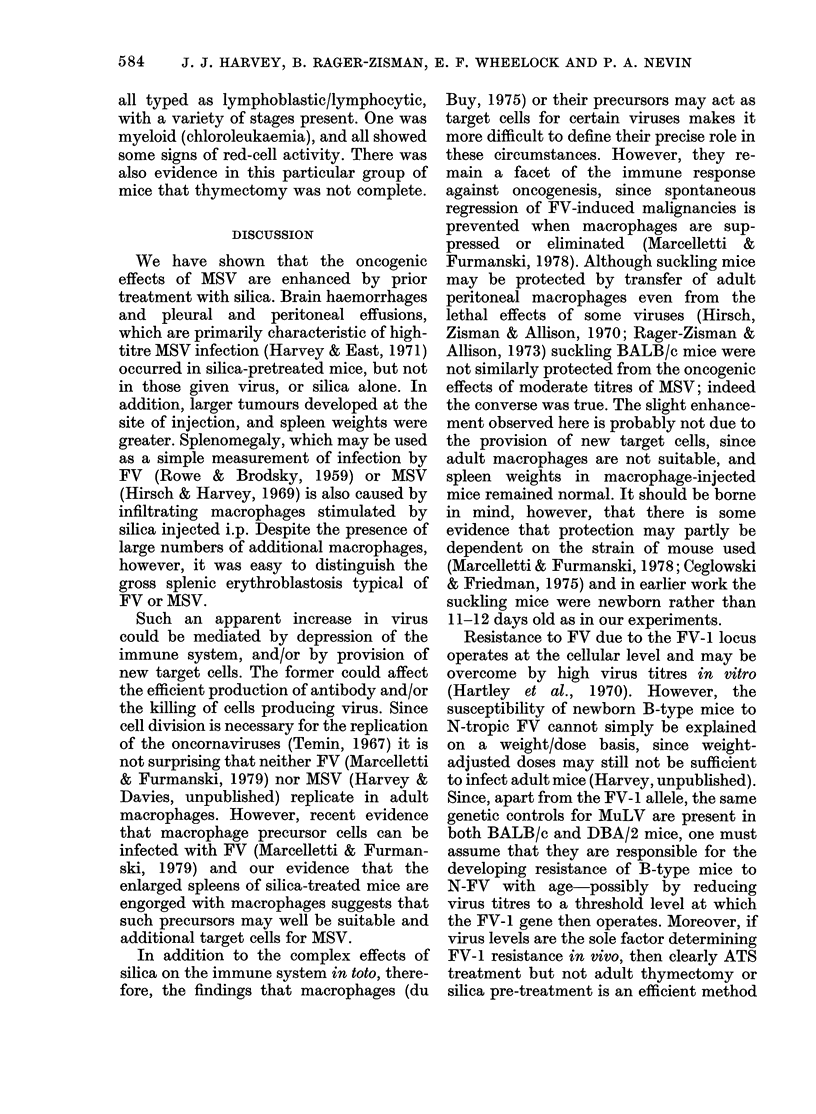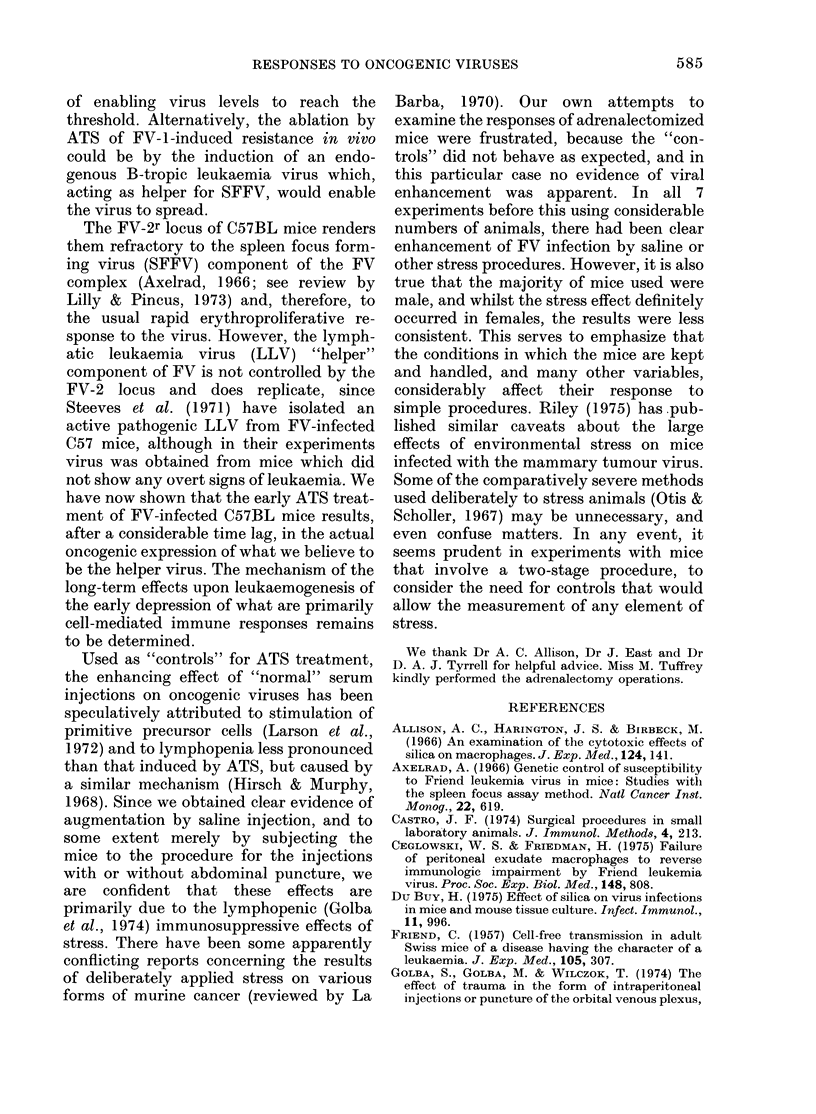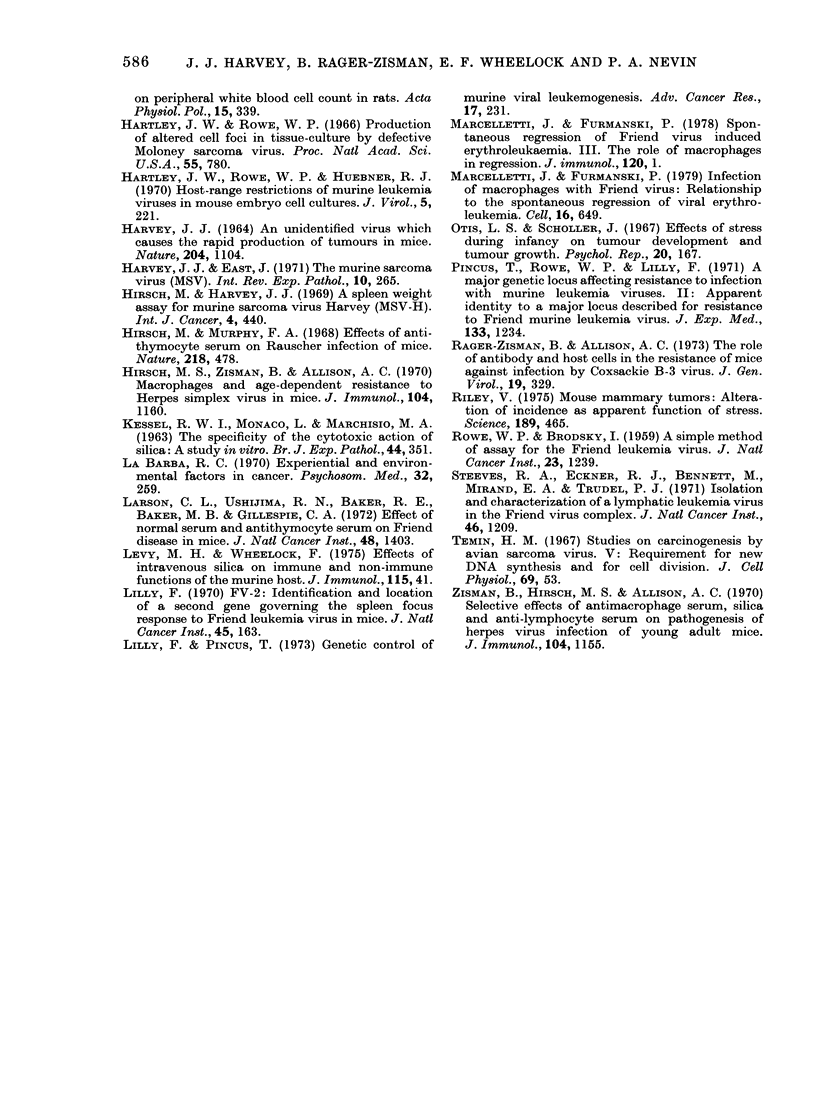# Factors affecting responses to murine oncogenic viral infections.

**DOI:** 10.1038/bjc.1980.100

**Published:** 1980-04

**Authors:** J. J. Harvey, B. Rager-Zisman, E. F. Wheelock, P. A. Nevin

## Abstract

**Images:**


*Br. J. Cancer* (1980) **41**, 577

### FACTORS AFFECTING RESPONSES TO MURINE ONCOGENIC VIRAL INFECTIONS

J. J. HARVEY, B. RAGER-ZISMAN, E. F. WHEELOCK AND P. A. NEVIN

*From the Division of Cell Pathology, MRC Clinical Research Centre, Harrow, Middlesex*

Received 10 September 1979 Accepted 23 November 1979

**Summary.—Silica specifically kills macrophages *in vitro*, and *in vivo* has been used as a method of determining the possible immunological or other roles of macrophages in a number of viral infections. In experiments reported here, injection of 30 or 50 mg silica i.p. increased the severity of the oncogenic effects of the murine sarcoma virus (MSV) and Friend virus (FV) in BALB/c mice. Unlike Herpes simplex and Coxsackie B-3 infections, however, passive transfer of adult macrophages to suckling mice did not protect the latter against MSV. In mice injected with silica, histological evidence of the compensatory proliferation of macrophages suggests that precursors of these cells may act as target cells for the virus and that this may override any immunosuppressive response effected by the silica. In addition, there was a considerable enhancing effect on the erythroproliferative response to both MSV and FV by injection of saline 5 h before the virus, and indeed to FV after only a simple abdominal needle puncture. We attributed this to the lymphopenic immunodepressive effects of stress, and our data may explain previously published findings of augmented oncogenic responses in mice after "normal" serum injections.**

**Newborn BALB/c (FV-$1^b$) mice were susceptible to N-tropic FV, but developed resistance by 29 days of age. Antithymocyte serum (ATS) but not silica injections or adult thymectomy ablated this resistance. C57BL (FV-$2^r$) mice were completely resistant to FV; however, those receiving FV and ATS developed late-onset leukaemia histologically characteristic of that produced by the helper component of the FV complex.**

Silica, administered i.p. or i.v., has been shown to exert a variety of influences on the immune system of the mouse, believed in some part to act *via* specific macrophage killing, which is readily demonstrable *in vitro* (Kessel *et al.*, 1963; Allison *et al.*, 1966; Levy & Wheelock, 1975). Some viral infections (*e.g.* Herpes simplex and Coxsackie B-3) cause severe effects in, and may be lethal to, suckling mice, whereas adults are resistant. The passive transfer of macrophages from adult mice protects susceptible sucklings, whilst conversely the resistance of adults is reduced or ablated by prior treatment with silica (Hirsch *et al.*, 1970; Rager-Zisman & Allison, 1973; Zisman *et al.*, 1970). Both these situations indicate a contributory role for the macrophage in immune protection against viral infections. However, the factors are complex *in vivo*, since macrophage damage by silica actually stimulates proliferation and migration of new macrophage populations. Some enhanced viral effects may be due, therefore, not solely to immunosuppression mediated by the killing of macrophages, but to increased viral replication in and distribution by this new population (du Buy, 1975).

We were interested to know whether the killing of macrophages *in vivo* by silica would alter the response to murine oncogenic viral infections. In particular, the murine sarcoma virus (MSV) (Harvey, 1964) loses most of its oncogenic potential in adult mice, and we tested whether treatment by silica before MSV had an enhancing effect, and whether transfer of adult macrophages would protect suckling mice. The erythroproliferative disease caused by Friend virus (FV) (Friend, 1957) is enhanced by prior treatment of adult mice with silica (Larson *et al.*, 1972) but passive transfer of macrophages may or may not protect (Marcelletti & Furmanski, 1978; Ceglowski & Friedman, 1975). However, unlike MSV, both suckling and adult mice are susceptible to FV.

In parallel with experiments in which we looked at the effect of silica on MSV and FV infections, we used DBA2 and BALB/c strains of mice, which have the same H2 complex and with the exception of the FV-1 locus (Lilly, 1970) similar genes controlling murine leukaemia virus (MuLV) infections, to explore the possibility that treatments such as silica, ATS or thymectomy might influence FV-1 control *in vivo*.

### METHODS AND MATERIALS

*Animals.*—BALB/c mice were bred at the Clinical Research Centre; DBA2 and C57BL strains were obtained from OLAC Ltd, Shaw's Farm, Bicester.

*Viruses.*—The origin of the Harvey strain of MSV has been described (Harvey, 1964). The virus used in these experiments was maintained by filtrate passage in BALB/c mice, and titrated by focus formation on mouse embryo fibroblasts (Hartley & Rowe, 1966).

Two strains of Friend virus were used, an "N-tropic" strain (FV-N) (Hartley *et al.*, 1970) was maintained by filtrate passage in DBA2 (N-type) mice. As expected, BALB/c (B-type) adult mice were resistant to FV-N (Pincus *et al.*, 1971). An "NB-tropic" strain (FV-NB), originally obtained from Dr C. Friend, was maintained by filtrate passage in BALB/c mice (the DBA2 strain was also susceptible). FV-N and FV-NB were titrated *in vivo* using the spleen-weight assay (Rowe & Brodsky, 1959).

*Silica.*—Silica, in the form of quartz dust (average particle size 5 $\mu$m), was obtained from Dowson & Dobson Ltd. Just before use, autoclaved dust was suspended in phosphate-buffered saline (PBS), or in Eagle's minimal essential medium (MEM) and exposed briefly to ultrasonic vibration.

*Macrophages.*—Peritoneal macrophages, washed from the abdominal cavities of adult mice with PBS at pH 7·1, were separated from other peritoneal cells by their adherence to plastic. "Stimulated" macrophages were prepared by inoculating mice i.p. with 2 ml of a 10% protease peptone solution 72 h before removing cells as described above.

*Antithymocyte serum (ATS).*—Thymuses from 6–8-week-old mice were gently pressed through a stainless-steel mesh. The resultant cell suspension was incubated at 37°C to remove adherent macrophages, washed twice and then passed through sterile cotton wool. Rabbits were injected i.v. twice with $10^8$–$10^9$ viable cells with a 10-day interval. Serum collected 10 days after the second immunization was absorbed twice for 1 h at room temperature with fresh, washed, mouse red blood cells (1:1) and then with cultured macrophages for a further hour. Serum was filtered through a 0·45 $\mu$m Millipore Swinnex filter before storage. Activity was measured *in vivo* by the retention by CBA mice of A-strain skin grafts for longer than 3 weeks.

*Adrenalectomy.*—Eight-week-old BALB/c mice, anaesthetized by Nembutal, were adrenalectomized according to the protocol of Castro (1974). Operated animals were then given a 1:1 saline/dextrose mixture to drink.

*Thymectomy.*—Thymus lobes were removed from 5-week-old mice anaesthetized with Nembutal, by aspiration with a water-jet vacuum pump *via* a midline anterior-mediastinal approach. At necropsy, thymectomized animals were screened histologically for evidence of any remaining thymic tissue.

*Histology.*—Tissues were fixed in formol-acetic alcohol and stained with haematoxylin and eosin. Organ imprints and blood smears were stained with May–Grünwald Giemsa.

### RESULTS

### *Effect of silica on the oncogenicity of MSV*

The first group of experiments was designed to see whether prior injection of silica suspension into BALB/c mice enhanced their response to MSV (Table I). A 30-mg silica suspension in MEM was injected i.p. into 4-week-old BALB/c mice, followed 24 h later by virus ($10^{4\cdot5}$ FFU/ml). We injected either 0·2 ml i.p. or

TABLE I.—*Enhancing effect of* 30 *mg i.p. silica on MSV infection in* 4-*week-old BALB/c mice**

| Treatment | Dose† and route of MSV | No. and sex injected | Mean spleen wt (mg) (and range) | Mean % PCV (and range) | No. with other MSV effects: Brain haemorrhages | Effusions‡ | Peritoneal tumours |
|---|---|---|---|---|---|---|---|
| MSV | 0·2 i.p. | 10 (5♂, 5♀) | 386 (120–1022) | 43 (39–49) | 0 | 0 | 0 |
| | 0·1 i.p. + 0·1 i.m. | 10 (6♂, 4♀) | 208 (144–351) | 43 (32–49) | 0 | 0 | 0 |
| Silica + MSV | 0·2 i.p. | 9 (3♂, 6♀) | 837 (632–1284) | 26 (13–48) | 4 | 6 | 3 |
| | 0·1 i.p. + 0·1 i.m. | 8 (3♂, 5♀) | 775 (280–1073) | 31 (24–39) | 0 | 6 | 2 |
| Silica | | 10 (5♂, 5♀) | 356 (169–533) | 42 (39–44) | 0 | 0 | 0 |
| None | | 20 (10♂, 10♀) | 138 (102–200) | 46 (43–49) | | | |

* All injected mice killed 16 days later, when 46 days old; uninjected mice at 43 days old.
† All at $10^{4.5}$ FFU/ml.
‡ Pleural and/or peritoneal.

the same dose divided between two routes, 0·1 ml i.p. and 0·1 ml i.m. into the thigh muscle. Sixteen days later the animals were killed, spleens were weighed and blood packed-cell volumes were measured in addition to the routine histology of affected tissues. The mice were compared with untreated controls, and others injected with MSV or silica only.

Mice injected with silica alone had obviously enlarged spleens, livers, mesenteric and parathymic lymph nodes, accompanied by massive mesenteric adhesions to the gut, liver, and peritoneal and diaphragm walls. Spleen weights were in some cases increased to 4 × normal, and histology showed that this was due mainly to accumulations of macrophages; follicles were also enlarged and disorganized and cell debris common. Evidence of increased haemopoiesis, fibrosis of the splenic capsule and considerable numbers of polymorphs were other variable features. No tumours were present.

Mice injected solely with MSV showed characteristic pathological effects, namely, splenomegaly due to erythroblastic proliferation, depressed blood packed-cell volumes, and one animal had a small sarcoma of the diaphragm wall. It should be noted that no mice developed tumours of the thigh; the age of the mice and dose of MSV were deliberately chosen to give only slight effects, since we were interested in possible enhancement by silica. As described in earlier reports (Harvey & East, 1971) the route of injection affected the pattern of response; mice injected i.p. had greater splenomegaly than those injected with the same dose divided between the i.p. and i.m. routes.

As can be seen in Table I, silica pretreatment drastically enhanced the pathological effects of MSV; spleen weights were greater, blood packed-cell volumes lower and, in contrast to those injected with either virus or silica alone, the majority (12/17) had massive pleural effusions, which in some cases were accompanied by pin-point brain haemorrhages (Figure). Pleural effusions, typically induced by high-titre MSV (Harvey strain) are rapidly lethal (Harvey & East, 1971); indeed, this determined the date the experiment was terminated, since only the mice in this group were dying. As in those mice injected with silica alone, the route of injection still exerted a clear effect on the resultant pathology. In the silica-pretreated group there were also more diaphragm and peritoneal tumours characteristic of i.p. MSV infection. Indeed, the number counted in this group may not reflect the true extent of the increase,

FIG.—Meningeal haemorrhage lying adjacent to the cerebellum of a BALB/c mouse injected with MSV and silica. Inflammatory cells are present within the haemorrhage. × 105.

since it was difficult to see small tumours because of the widespread adhesions caused by silica. It was interesting that enhancement, while obvious, was not such that tumours arose at the site of i.m. injection, although many mice were extremely sick when killed only 16 days after injection. Spleens were enlarged in the groups injected with silica or MSV only, but the effect of combined treatment was more than additive, particularly in those injected with virus divided between two routes. However, in this dually treated group the histological appearance was generally that of a response to higher titre MSV (*i.e.* much increased haemopoeisis) rather than an equal mixture of this effect and obvious macrophage infiltration. If the enhancement of MSV by silica was mediated *via* a depression of the immune response caused by the killing of macrophages, then it was possible, as has been shown for other viral infections (Hirsch *et al.*, 1970; Rager-Zisman & Allison, 1973) that adult macrophages could exert a protective effect against MSV in suckling mice.

*Failure of transferred adult peritoneal macrophages to protect suckling mice against MSV*

Either $2 \times 10^7$ stimulated or unstimulated adult BALB/c macrophages suspended in 0·1 ml MEM, or 0·1 ml MEM alone, were injected i.p. into 11–12-day-old BALB/c mice 5 h before MSV. Controls were age-matched untreated mice, and those receiving virus or macrophages only. Mice in most affected groups rapidly became sick, and all were killed 13 days after infection. Table II shows that neither stimulated nor unstimulated adult BALB/c macrophages protected suckling BALB/c mice from the effects of MSV; indeed, on the contrary, the effects were enhanced. Spleen weights and tumour sizes at the site of i.m. injection were greater, and anaemia more severe. It

TABLE II.—*Enhancing effect on MSV of* $2 \times 10^7$ *adult peritoneal macrophages injected i.p. into suckling* (11–12 *days old*)*BALB/c mice** 5 *h before virus*

| Treatment | No. and sex injected | Spleen wt (mg) | % PCV | No. of tumours: i.p. | No. of tumours: Diaphragm and splenic mesentery | Diam. of i.m. tumour (mm) |
|---|---|---|---|---|---|---|
| MSV‡ | 23 (12♂, 11♀) | 184 (114–270) | 39 (37–45) | 21 | 9 | 5·9 (5·5–7·5) |
| Macrophages +MSV | 15 (8♂, 7♀) | 307 (145–609) | 36 (22–41) | 28 | 21 | 7·9 (5·5–11) |
| "Stimulated" macrophages +MSV | 14 (18♂, 6♀) | 317 (186–518) | 32 (21–38) | 29 | 18 | 9·5 (4·5–12) |
| MEM+MSV | 9 (5♂, 4♀) | 235 (147–459) | 40·5 (30–49) | ND | | ND |
| Stimulated macrophages | 4 (2♂, 2♀) | 109 (100–121) | ND | | | |
| None (28 days old) | 20 (10♂, 10♀) | 126 (99–161) | 42 (35–50) | | | |

* All mice killed 13 days after infection (24 days old).
‡ Titre = $10^{4\cdot5}$ FFU/ml, dose = 0·1 ml i.p. + 0·05 ml i.m. (thigh).

should be noted that at the age that the mice were killed (24 days) a leg width of 9 mm represents gross enlargement. There were more tumours in the splenic mesentery and diaphragm of macrophage-pretreated groups than in the controls, although no clear difference was seen in the numbers disseminated at other sites throughout the peritoneum. There was also some evidence of enhancement of MSV in the group preinjected with MEM only, although in this case data on thigh tumour sizes were not obtained. No mice developed pleural or peritoneal effusions or brain haemorrhages.

*Enhancing effects of silica and stress on FV infection*

In parallel with these experiments, we confirmed an earlier report (Larson *et al.*, 1972) that silica pretreatment enhances the effects of Friend virus, which is oncogenic in both adult and suckling mice. Thirty or 50 mg silica suspended in 0·5 ml MEM, or 0·5 ml MEM alone, were injected i.p. into adult female BALB/c mice, followed 5 h later by 0·1 ml FV-NB. Control groups were injected with silica or virus alone. Spleen weights were obtained 19 days later. Typical erythroblastic splenomegaly occurred in the mice injected with FV only. 50 mg silica only caused the same widespread effects described in weanlings in the previous experiment, whereas those in mice injected with 30 mg were far less severe. Table III shows a clear dose–response relationship between silica and spleen weights. Both 30 and 50 mg silica enhanced the erythroblastic effect of FV, and augmentation of

TABLE III.—*Enhancing effect of silica injected i.p. on FV-NB infection in adult BALB/c female mice**

| Treatment | No. injected | Spleen wt (mg) |
|---|---|---|
| FV† | 8 | 437 (134–1206) |
| Silica (50 mg) | 5 | 555 (515–599) |
| Silica (50 mg) +FV | 5 | 2277 (2197–2902) |
| Silica (30 mg) | 6 | 370 (329–407) |
| Silica (30 mg) +FV | 5 | 1962 (476–3613) |
| MEM+FV | 5 | 1199 (647–1981) |
| None | 10 | 120 (101–138) |

* All killed 19 days after injection.

† Dose = $10^3$ $ID_{50}$/mouse.

spleen weights in groups receiving both virus and silica was more than the sum of that caused by either the virus, or respective doses of silica alone.

Although there had been an indication that pre-treatment with MEM had slightly enhanced the response to MSV (Table II) we were surprised to find how greatly it affected the FV-infected mice. This experiment (Table III) was therefore repeated using PBS or MEM, both of which had been used as suspending media for silica in earlier experiments (Table IV). Both 5h and 18h intervals between injections were tested. There was, again, enhancement of erythroblastic splenomegaly in animals pretreated with saline or MEM, and this was greater when injections were 5 rather than 18 h apart. It seemed possible that MEM or PBS contained factors which stimulated production of target cells for the virus. In further experiments, therefore, we included "sham-injected" groups which received an abdominal needle puncture but no fluid. Again (Table V), spleen weights in pretreated animals were greater, whether or not fluid had been injected before the virus.

TABLE IV.—*Enhancement of FV-NB induced splenomegaly in 20-week-old BALB/c mice† by prior i.p. injection of 0·2 ml PBS or MEM*

| Treatment | No. and sex injected | Mean spleen wt (mg) |
|---|---|---|
| FV* | 12 (6♂, 6♀) | 477 (215–704) |
| PBS or MEM + FV (5 h later) | 12 (6♂, 6♀) | 1180 (424–2300) |
| PBS or MEM + FV (18 h later) | 16 (8♂, 8♀) | 783 (211–1891) |
| PBS or MEM | 12 (6♂, 6♀) | 158 (129–202) |

* Dose = $10^{2\cdot5}ID_{50}$/mouse.

† All killed 21 days after injection.

TABLE V.—*Enhancement of FV-NB induced splenomegaly in 8-week-old BALB/c male mice† by prior i.p. injection of 0·2 ml PBS, and by simple abdominal needle puncture*

| Treatment | No. injected | Mean spleen wt (mg) |
|---|---|---|
| FV* | 9 | 739 (411–1237) |
| PBS + FV (5 h later) | 10 | 1217 (826–1622) |
| Abdominal needle puncture + FV (5 h later) | 10 | 1154 (409–1964) |

* Dose = $10^3ID_{50}$/mouse.

† All killed 21 days after injection.

Thus it became clear that some enhancement might be due to stress—possibly *via* an effect on the immune system by adrenal corticosteroid hormone. Adrenalectomized and intact female mice (10 per group) were therefore given FV, but showed comparable spleen weights 3 weeks later (means of 873 and 872 mg respectively) although the former were slightly more anaemic. Unexpectedly, saline pretreatment did not obviously enhance the splenic response to FV, in either adrenalectomized or intact mice (mean weights 906 and 995 mg respectively), so no conclusions could be drawn regarding any stress effect mediated by adrenal corticosteroid hormone. In these experiments, all the animals, including relevant controls, had been held in the same animal room for several weeks before injection, whereas previously mice had been removed from the relatively quiet SPF breeding unit to the experimental rooms just before use. It was possible that the more "settled" animals had not been stressed by the injection procedure. Nevertheless, when we then compared mice reared in the experimental animal laboratory with others bred and recently transferred from the SPF unit, pretreatment of whatever kind 5 h before FV again had an enhancing effect on the virus (Table VI). This included animals simply taken out of their cages and immediately replaced. Whilst enhancement was particularly evident in the SPF males, those conventionally reared were more variable in response. The same trend occurred in female mice, but spleen weights were too variable for a clear conclusion to be reached.

TABLE VI.—*Comparison of response to "stress" on FV-NB-infection in specific pathogen free (SPF) and conventionally reared (CR) BALB/c mice**

| Treatment | Male Mean spleen wt (mg) SPF | CR | Female Mean spleen wt (mg) SPF | CR |
|---|---|---|---|---|
| FV‡ | 488 (212–792) | 692 (327–920) | 1460 (535–2114) | 1473 (207–2407) |
| PBS + FV (5 h later) | 853 (418–1379) | 850 (416–1150) | 1223 (562–1780) | 1844 (1534–2419) |
| "Sham" injection + FV (5 h later) | 876 (456–1543) | 1017† (272–2211) | 1822 (844–2657) | 2079 (1244–2798) |
| "Handling" + FV (5 h later) | 764 (202–1156) | 479† (343–791) | 1716 (427–2586) | 1608 (706–2625) |
| PBS | 125 (114–143) | 145 (129–166) | 129 (123–144) | 150 (125–192) |

* All killed 20 days after injection.

‡ Dose = $10^3 ID_{50}$/mouse.

5 mice per group, except groups marked† (6).

*Factors affecting the resistance of mice to FV*

Concurrently we examined the resistance of BALB/c ("B-type") mice to a strain of N-tropic FV which is lethal in adult DBA/2 ("N-type") mice. The resistance of BALB/c mice to N-tropic FV is age-dependent. All 24 mice tested were susceptible to $10^4$ $ID_{50}$, if injected when they were 6 days old or less. By 11 days, although most (7/8) still developed erythroblastic splenomegaly, latent periods had increased, and one individual remained healthy for the duration of the experiment (38 weeks). An increasing number (13/22) were resistant when injected at 14–21 days, and by 29 days resistance was complete. Since, therefore, resistance to FV determined by the FV-1 locus is not absolute *in vivo*, we tested whether it could be abrogated in adult mice by various treatments affecting the immune response such as silica, ATS and/or thymectomy.

Resistance of BALB/c adults to FV-N was not affected by injecting 50 mg silica 5 h before the virus, nor by adult thymectomy. By contrast, ATS completely abrogated the resistance of either adult intact or thymectomized BALB/c mice to N-tropic FV; all recipients of FV and ATS treatment rapidly developed erythroblastic splenomegaly (Table VII).

To investigate further the effect of ATS treatment, C57BL mice were tested. These are usually completely refractory to FV, due to the presence of the $FV2^r$ gene, and are also a "B-type" strain with regard to their sensitivity to N- and B-tropic FV (Pincus *et al.*, 1971). Even so, they developed late-onset leukaemia although thymectomized as adults, treated with ATS and injected with $10^4$ $ID_{50}$ N-tropic FV. In one experiment, 5/7 C57BL mice become leukaemic after 31–61 weeks, but an intact group only given virus remained healthy throughout the same 61 weeks. Histologically, the leukaemias were nearly

TABLE VII.—*Ablation by ATS treatment of the resistance of adult female BALB/c mice to N-tropic FV*

| Treatment | Latent period (days) | No. with EBS†/No. injected | Mean spleen wt (mg) |
|---|---|---|---|
| FV-N* | 107 | 0/6 | 133 (109–149) |
| FV-N + ATS‡ | 26 | 8/8 | 1560 (460–2125) |
| FV-N + TX§ | 180 | 0/7 | 174 (132–297) |
| FV-N + ATS + TX | 25 | 8/8 | 1750 (920–2452) |

* Dose = $10^4 ID_{50}$/mouse.
† EBS = erythroblastic splenomegaly.
‡ Starting when mice were 8 weeks old, 5 consecutive daily injections of ATS, the 4th coincident with FV.
§ Thymectomy at 5 weeks.

all typed as lymphoblastic/lymphocytic, with a variety of stages present. One was myeloid (chloroleukaemia), and all showed some signs of red-cell activity. There was also evidence in this particular group of mice that thymectomy was not complete.

### DISCUSSION

We have shown that the oncogenic effects of MSV are enhanced by prior treatment with silica. Brain haemorrhages and pleural and peritoneal effusions, which are primarily characteristic of high-titre MSV infection (Harvey & East, 1971) occurred in silica-pretreated mice, but not in those given virus, or silica alone. In addition, larger tumours developed at the site of injection, and spleen weights were greater. Splenomegaly, which may be used as a simple measurement of infection by FV (Rowe & Brodsky, 1959) or MSV (Hirsch & Harvey, 1969) is also caused by infiltrating macrophages stimulated by silica injected i.p. Despite the presence of large numbers of additional macrophages, however, it was easy to distinguish the gross splenic erythroblastosis typical of FV or MSV.

Such an apparent increase in virus could be mediated by depression of the immune system, and/or by provision of new target cells. The former could affect the efficient production of antibody and/or the killing of cells producing virus. Since cell division is necessary for the replication of the oncornaviruses (Temin, 1967) it is not surprising that neither FV (Marcelletti & Furmanski, 1979) nor MSV (Harvey & Davies, unpublished) replicate in adult macrophages. However, recent evidence that macrophage precursor cells can be infected with FV (Marcelletti & Furmanski, 1979) and our evidence that the enlarged spleens of silica-treated mice are engorged with macrophages suggests that such precursors may well be suitable and additional target cells for MSV.

In addition to the complex effects of silica on the immune system *in toto*, therefore, the findings that macrophages (du Buy, 1975) or their precursors may act as target cells for certain viruses makes it more difficult to define their precise role in these circumstances. However, they remain a facet of the immune response against oncogenesis, since spontaneous regression of FV-induced malignancies is prevented when macrophages are suppressed or eliminated (Marcelletti & Furmanski, 1978). Although suckling mice may be protected by transfer of adult peritoneal macrophages even from the lethal effects of some viruses (Hirsch, Zisman & Allison, 1970; Rager-Zisman & Allison, 1973) suckling BALB/c mice were not similarly protected from the oncogenic effects of moderate titres of MSV; indeed the converse was true. The slight enhancement observed here is probably not due to the provision of new target cells, since adult macrophages are not suitable, and spleen weights in macrophage-injected mice remained normal. It should be borne in mind, however, that there is some evidence that protection may partly be dependent on the strain of mouse used (Marcelletti & Furmanski, 1978; Ceglowski & Friedman, 1975) and in earlier work the suckling mice were newborn rather than 11–12 days old as in our experiments.

Resistance to FV due to the FV-1 locus operates at the cellular level and may be overcome by high virus titres *in vitro* (Hartley *et al.*, 1970). However, the susceptibility of newborn B-type mice to N-tropic FV cannot simply be explained on a weight/dose basis, since weight-adjusted doses may still not be sufficient to infect adult mice (Harvey, unpublished). Since, apart from the FV-1 allele, the same genetic controls for MuLV are present in both BALB/c and DBA/2 mice, one must assume that they are responsible for the developing resistance of B-type mice to N-FV with age—possibly by reducing virus titres to a threshold level at which the FV-1 gene then operates. Moreover, if virus levels are the sole factor determining FV-1 resistance *in vivo*, then clearly ATS treatment but not adult thymectomy or silica pre-treatment is an efficient method of enabling virus levels to reach the threshold. Alternatively, the ablation by ATS of FV-1-induced resistance *in vivo* could be by the induction of an endogenous B-tropic leukaemia virus which, acting as helper for SFFV, would enable the virus to spread.

The FV-$2^r$ locus of C57BL mice renders them refractory to the spleen focus forming virus (SFFV) component of the FV complex (Axelrad, 1966; see review by Lilly & Pincus, 1973) and, therefore, to the usual rapid erythroproliferative response to the virus. However, the lymphatic leukaemia virus (LLV) "helper" component of FV is not controlled by the FV-2 locus and does replicate, since Steeves *et al.* (1971) have isolated an active pathogenic LLV from FV-infected C57 mice, although in their experiments virus was obtained from mice which did not show any overt signs of leukaemia. We have now shown that the early ATS treatment of FV-infected C57BL mice results, after a considerable time lag, in the actual oncogenic expression of what we believe to be the helper virus. The mechanism of the long-term effects upon leukaemogenesis of the early depression of what are primarily cell-mediated immune responses remains to be determined.

Used as "controls" for ATS treatment, the enhancing effect of "normal" serum injections on oncogenic viruses has been speculatively attributed to stimulation of primitive precursor cells (Larson *et al.*, 1972) and to lymphopenia less pronounced than that induced by ATS, but caused by a similar mechanism (Hirsch & Murphy, 1968). Since we obtained clear evidence of augmentation by saline injection, and to some extent merely by subjecting the mice to the procedure for the injections with or without abdominal puncture, we are confident that these effects are primarily due to the lymphopenic (Golba *et al.*, 1974) immunosuppressive effects of stress. There have been some apparently conflicting reports concerning the results of deliberately applied stress on various forms of murine cancer (reviewed by La Barba, 1970). Our own attempts to examine the responses of adrenalectomized mice were frustrated, because the "controls" did not behave as expected, and in this particular case no evidence of viral enhancement was apparent. In all 7 experiments before this using considerable numbers of animals, there had been clear enhancement of FV infection by saline or other stress procedures. However, it is also true that the majority of mice used were male, and whilst the stress effect definitely occurred in females, the results were less consistent. This serves to emphasize that the conditions in which the mice are kept and handled, and many other variables, considerably affect their response to simple procedures. Riley (1975) has published similar caveats about the large effects of environmental stress on mice infected with the mammary tumour virus. Some of the comparatively severe methods used deliberately to stress animals (Otis & Scholler, 1967) may be unnecessary, and even confuse matters. In any event, it seems prudent in experiments with mice that involve a two-stage procedure, to consider the need for controls that would allow the measurement of any element of stress.

We thank Dr A. C. Allison, Dr J. East and Dr D. A. J. Tyrrell for helpful advice. Miss M. Tuffrey kindly performed the adrenalectomy operations.

### REFERENCES

Allison, A. C., Harington, J. S. & Birbeck, M. (1966) An examination of the cytotoxic effects of silica on macrophages. *J. Exp. Med.*, **124**, 141.

Axelrad, A. (1966) Genetic control of susceptibility to Friend leukemia virus in mice: Studies with the spleen focus assay method. *Natl Cancer Inst. Monog.*, **22**, 619.

Castro, J. F. (1974) Surgical procedures in small laboratory animals. *J. Immunol. Methods*, **4**, 213.

Ceglowski, W. S. & Friedman, H. (1975) Failure of peritoneal exudate macrophages to reverse immunologic impairment by Friend leukemia virus. *Proc. Soc. Exp. Biol. Med.*, **148**, 808.

Du Buy, H. (1975) Effect of silica on virus infections in mice and mouse tissue culture. *Infect. Immunol.*, **11**, 996.

Friend, C. (1957) Cell-free transmission in adult Swiss mice of a disease having the character of a leukaemia. *J. Exp. Med.*, **105**, 307.

Golba, S., Golba, M. & Wilczok, T. (1974) The effect of trauma in the form of intraperitoneal injections or puncture of the orbital venous plexus, on peripheral white blood cell count in rats. *Acta Physiol. Pol.*, **15**, 339.

HARTLEY, J. W. & ROWE, W. P. (1966) Production of altered cell foci in tissue-culture by defective Moloney sarcoma virus. *Proc. Natl Acad. Sci. U.S.A.*, **55**, 780.

HARTLEY, J. W., ROWE, W. P. & HUEBNER, R. J. (1970) Host-range restrictions of murine leukemia viruses in mouse embryo cell cultures. *J. Virol.*, **5**, 221.

HARVEY, J. J. (1964) An unidentified virus which causes the rapid production of tumours in mice. *Nature*, **204**, 1104.

HARVEY, J. J. & EAST, J. (1971) The murine sarcoma virus (MSV). *Int. Rev. Exp. Pathol.*, **10**, 265.

HIRSCH, M. & HARVEY, J. J. (1969) A spleen weight assay for murine sarcoma virus Harvey (MSV-H). *Int. J. Cancer*, **4**, 440.

HIRSCH, M. & MURPHY, F. A. (1968) Effects of antithymocyte serum on Rauscher infection of mice. *Nature*, **218**, 478.

HIRSCH, M. S., ZISMAN, B. & ALLISON, A. C. (1970) Macrophages and age-dependent resistance to Herpes simplex virus in mice. *J. Immunol.*, **104**, 1160.

KESSEL, R. W. I., MONACO, L. & MARCHISIO, M. A. (1963) The specificity of the cytotoxic action of silica: A study *in vitro*. *Br. J. Exp. Pathol.*, **44**, 351.

LA BARBA, R. C. (1970) Experiential and environmental factors in cancer. *Psychosom. Med.*, **32**, 259.

LARSON, C. L., USHIJIMA, R. N., BAKER, R. E., BAKER, M. B. & GILLESPIE, C. A. (1972) Effect of normal serum and antithymocyte serum on Friend disease in mice. *J. Natl Cancer Inst.*, **48**, 1403.

LEVY, M. H. & WHEELOCK, F. (1975) Effects of intravenous silica on immune and non-immune functions of the murine host. *J. Immunol.*, **115**, 41.

LILLY, F. (1970) FV-2: Identification and location of a second gene governing the spleen focus response to Friend leukemia virus in mice. *J. Natl Cancer Inst.*, **45**, 163.

LILLY, F. & PINCUS, T. (1973) Genetic control of murine viral leukemogenesis. *Adv. Cancer Res.*, **17**, 231.

MARCELLETTI, J. & FURMANSKI, P. (1978) Spontaneous regression of Friend virus induced erythroleukaemia. III. The role of macrophages in regression. *J. immunol.*, **120**, 1.

MARCELLETTI, J. & FURMANSKI, P. (1979) Infection of macrophages with Friend virus: Relationship to the spontaneous regression of viral erythroleukemia. *Cell*, **16**, 649.

OTIS, L. S. & SCHOLLER, J. (1967) Effects of stress during infancy on tumour development and tumour growth. *Psychol. Rep.*, **20**, 167.

PINCUS, T., ROWE, W. P. & LILLY, F. (1971) A major genetic locus affecting resistance to infection with murine leukemia viruses. II: Apparent identity to a major locus described for resistance to Friend murine leukemia virus. *J. Exp. Med.*, **133**, 1234.

RAGER-ZISMAN, B. & ALLISON, A. C. (1973) The role of antibody and host cells in the resistance of mice against infection by Coxsackie B-3 virus. *J. Gen. Virol.*, **19**, 329.

RILEY, V. (1975) Mouse mammary tumors: Alteration of incidence as apparent function of stress. *Science*, **189**, 465.

ROWE, W. P. & BRODSKY, I. (1959) A simple method of assay for the Friend leukemia virus. *J. Natl Cancer Inst.*, **23**, 1239.

STEEVES, R. A., ECKNER, R. J., BENNETT, M., MIRAND, E. A. & TRUDEL, P. J. (1971) Isolation and characterization of a lymphatic leukemia virus in the Friend virus complex. *J. Natl Cancer Inst.*, **46**, 1209.

TEMIN, H. M. (1967) Studies on carcinogenesis by avian sarcoma virus. V: Requirement for new DNA synthesis and for cell division. *J. Cell Physiol.*, **69**, 53.

ZISMAN, B., HIRSCH, M. S. & ALLISON, A. C. (1970) Selective effects of antimacrophage serum, silica and anti-lymphocyte serum on pathogenesis of herpes virus infection of young adult mice. *J. Immunol.*, **104**, 1155.